# Supplementary material for: Evaluation of the Intrinsic and Perceived Quality of Sangiovese Wines from California and Italy
Source: Foods. 2020 Aug 10;9(8):1088. doi: 10.3390/foods9081088 (PMC7466209; doi:10.3390/foods9081088)
Supplement: Supplementary file 1 [file foods-09-01088-s001.pdf]

**Table S1.** Polyphenol composition of wines and color indices with the corresponding main effect significance levels (95%) and the averages (mg/L).

| compound n. | Compound | Italy | California | *p - value regions* | *p - value replicates* |
|---|---|---|---|---|---|
| 1 | **delphinidin-3-*O*-glucoside** | 17.81 a | 14.81 a | 0.0630 | 0.9995 |
| 2 | **cyanidin-3-*O*-glucoside** | 15.77 b | 8.12 a | 0.0000 | 0.9996 |
| 3 | **petunidin-3-*O*-glucoside** | 22.12 a | 23.74 b | 0.4608 | 1.0000 |
| 4 | **peonidin-3-*O*-glucoside** | 17.15 b | 13.02 a | 0.0070 | 0.9987 |
| 5 | **malvidin-3-*O*-glucoside** | 60.21 a | 77.95 b | 0.0166 | 0.9997 |
| 6 | **(+)-catechin** | 70.35 b | 24.67 a | 0.0000 | 0.7652 |
| 7 | **(-)-epicatechin** | 70.35 b | 28.57 a | 0.0000 | 0.9714 |
| 8 | **gallic acid** | 143.22 b | 52.01 a | 0.0000 | 0.9689 |
| 9 | **caftaric Acid** | 47.13 b | 38.54 a | 0.0243 | 0.9989 |
| 10 | **caffeic Acid** | 5.24 a | 5.00 a | 0.8971 | 0.9990 |
| 11 | **coutaric acid** | 10.06 b | 7.25 a | 0.0005 | 0.9968 |
| 12 | ***p*-coumaric acid** | 1.21 a | 1.51 a | 0.4133 | 0.9998 |
| 13 | **myrcetin-3-*O*-glucoside** | 17.45 a | 37.52 b | 0.0000 | 0.9411 |
| 14 | **myricetin** | 7.73 b | 1.78 a | 0.0000 | 0.9905 |
| 15 | **quercetin-3-*O*-galactoside** | 4.12 a | 6.71 b | 0.0020 | 0.9988 |
| 16 | **quercetin-3-*O*-glucoside** | 4.77 a | 69.01 b | 0.0000 | 0.9866 |
| 17 | **quercetin-3-*O*-glucuronide** | 40.34 a | 59.06 b | 0.0002 | 0.9441 |
| 18 | **quercetin-3-*O*-rhamnoside** | 1.83 a | 5.13 b | 0.0000 | 0.9927 |
| 19 | **quercetin** | 32.46 b | 11.21 a | 0.0000 | 0.9998 |
| 20 | **polymeric phenols** | 802.60 b | 302.71 a | 0.0000 | 0.2866 |
| 21 | **pigmented polymers** | 289.15 a | 233.15 a | 0.5951 | 0.3704 |
| 22 | **color intensity** | 11.67 b | 7.24 a | 0.0000 | 0.9999 |
| 23 | **hue** | 0.68 a | 0.82 b | 0.0000 | 0.9997 |
| 24 | **total phenols index** | 62.39 b | 45.21 a | 0.0000 | 1.0000 |

Different letters within the same row indicate significant differences

**Table S2.** Volatile compounds measured in the HS-SPME-GC-MS, with the corresponding main effect significance levels (95%) and the averages (mg/L).

| compound n. | Compound | Italy | California | *p - value regions* | *p - value replicates* |
|---|---|---|---|---|---|
| 1 | **ethyl acetate** | 20.56 a | 29.19 b | 0.0000 | 0.9931 |
| 2 | **isobuthylacetate** | 0.044 a | 0.098 b | 0.0000 | 0.9983 |
| 3 | **ethyl butanoate** | 0.052 a | 0.095 b | 0.0000 | 0.9986 |
| 4 | **propan-1-ol** | 1.619 a | 2.862 b | 0.0000 | 0.9339 |
| 5 | **ethyl-2-methylbutirate** | 0.008 a | 0.016 b | 0.0000 | 0.9688 |
| 6 | **ethylisovalerate** | 0.012 a | 0.018 b | 0.0000 | 0.9903 |
| 7 | **2-methylpropan-1-ol** | 19.14 a | 22.38 a | 0.0697 | 0.9518 |
| 8 | **3-methylbutyl acetate** | 2.33 a | 7.99 b | 0.0000 | 0.9981 |
| 9 | **butan-1-ol**[1] | 0.0017 a | 0.0025 b | 0.0001 | 0.9612 |
| 10 | **3-methylbutan-1-ol** | 73.54 a | 81.57 b | 0.0731 | 0.9063 |
| 11 | **ethyl hexanoate** | 0.484 a | 0.634 b | 0.0466 | 0.9959 |
| 12 | **pentan-1-ol**[1] | 0.0003 a | 0.0004 b | 0.0005 | 0.8684 |
| 13 | **isoamylbutanoate (isoamylacetate)**[1] | 0.0007 a | 0.0011 b | 0.0094 | 0.9834 |
| 14 | **hexyl acetate** | 0.0038 a | 0.0297 b | 0.0003 | 0.9897 |
| 15 | **octan-2-one1** | 0.0014 a | 0.0019 b | 0.1501 | 0.1952 |
| 16 | **3-methylpenthan-1-ol**[1] | 0.0009 a | 0.0012 b | 0.0155 | 0.7927 |
| 17 | **ethyl eptanoate** | 0.0017 a | 0.0038 b | 0.0000 | 0.8874 |
| 18 | **ethyl 2-hydroxypropanoate (ethyl lactate)** | 10.61 a | 15.49 b | 0.0011 | 0.8188 |
| 19 | **hexan-1-ol** | 1.32 a | 2.24 b | 0.0000 | 0.9728 |
| 20 | **methyl octanoate**[1] | 0.004 a | 0.004 b | 0.9883 | 0.8380 |
| 21 | **ethyl octanoate** | 0.246 a | 0.371 b | 0.0000 | 0.9833 |
| 22 | **1-octen-3-olo**[1] | 0.001 a | 0.002 b | 0.0867 | 0.1358 |
| 23 | **isoamyl hexanoate**[1] | 0.012 a | 0.012 b | 0.8402 | 0.9972 |
| 24 | **(2R,5R)-2,6,6-trimethyl-10-methylidene-1-oxaspiro[4.5]dec-8-ene (Vitispirane I)**[1] | 0.0013 a | 0.0019 b | 0.0094 | 0.9293 |
| 25 | **riesling acetale**[1] | 0.010 a | 0.014 b | 0.0005 | 0.8834 |
| 26 | **ethyl nonanoate** | 0.0010 a | 0.0014 b | 0.0041 | 0.8537 |
| 27 | **β-linalool** | 0.032 b | 0.027 a | 0.0001 | 0.7881 |
| 28 | **octan-1-ol** | 0.079 b | 0.060 a | 0.0047 | 0.9898 |
| 29 | **methyl decanoate**[1] | 0.0039 b | 0.0036 a | 0.4645 | 0.9535 |
| 30 | **4-terpineol**[1] | 0.00033 b | 0.000063 a | 0.0000 | 0.9472 |
| 31 | **ethyl decanoate** | 7.82 a | 10.02 b | 0.0137 | 0.9886 |
| 32 | **isoamyl octanoate**[1] | 0.0157 a | 0.0224 b | 0.003 | 0.9409 |
| 33 | **nonan-1-ol**[1] | 0.0062 a | 0.0064 a | 0.7876 | 0.6852 |
| 34 | **diethyl butanedioate (diethyl succinate)** | 5.65 a | 6.12 b | 0.7167 | 0.9901 |
| 35 | **ethyl dec-9-enoate**[1] | 0.0235 a | 0.0981 b | 0.0006 | 1.0000 |
| 36 | **α-terpineol**[1] | 0.00088 b | 0.00045 a | 0.0046 | 0.6516 |
| 37 | **(6E)-7,11-dimethyl-3-methylidenedodeca-1,6,10-triene (β-farnesene)**[1] | 0.0019 a | 0.0022 b | 0.5862 | 0.9850 |
| 38 | **ethyl undecanoate** | 0.0015 a | 0.0015 b | 0.6060 | 0.8552 |
| 39 | **1,1,6-trimethyl-1,2-dihydronaphthalene (TDN)**[1] | 0.00099 a | 0.00121 b | 0.3254 | 0.9711 |

| | | | | | |
|---|---|---|---|---|---|
| 40 | **α-farnesene[1]** | 0.00080 a | 0.00087 a | 0.4853 | 0.8571 |
| 41 | **decan-1-ol[1]** | 0.0061 a | 0.0063 b | 0.7092 | 0.4257 |
| 42 | **3,7-dimethyloct-6-en-1-ol (β-citronellol)** | 0.0454 a | 0.0451 a | 0.8383 | 0.9715 |
| 43 | **metilsalicilate[1]** | 0.00076 b | 0.00050 a | 0.0013 | 0.9526 |
| 44 | **geraniol[1]** | 0.000 a | 0.00056 b | 0.0294 | 0.9905 |
| 45 | **2-phenylethyl acetate (β-phenethyl acetate)** | 0.0616 a | 0.1164 b | 0.0122 | 0.9991 |
| 46 | **(E)-1-(2,6,6-trimethylcyclohexa-1,3-dien-1-yl)but-2-en-1-one (β-damascenone)** | 0.119 a | 0.126 b | 0.5686 | 0.9994 |
| 47 | **ethyl dodecanoate** | 0.0124 a | 0.0241 b | 0.0009 | 0.9898 |
| 48 | **nerylacetone[1]** | 0.00064 a | 0.00060 a | 0.7712 | 0.4953 |
| 49 | **isoamyl decanoate[1]** | 0.0046 a | 0.0074 b | 0.0058 | 0.9809 |
| 50 | **1,7-Octadien-3-ol, 2,6-dimethyl-[1]** | 0.0014 a | 0.0059 b | 0.0002 | 0.9997 |
| 51 | **butanedioic acid, ethyl 3-methylbutyl ester[1]** | 0.0249 a | 0.0194 a | 0.1951 | 0.9847 |
| 52 | **2-phenylethanol (β-phenylethanol)** | 19.44 b | 13.65 a | 0.0004 | 0.9024 |
| 53 | **(±)-trans-nerolidol[1]** | 0.0074 b | 0.0056 a | 0.0165 | 0.9842 |
| 54 | **octanoic acid** | 1.656 a | 2.447 b | 0.0000 | 0.8448 |
| 55 | **nonanoic acid[1]** | 0.0133 a | 0.0124 a | 0.4839 | 0.9308 |
| 56 | **decanoic acid[1]** | 0.0907 a | 0.0859 a | 0.5617 | 0.9211 |
| 57 | **dodecanoic acid[1]** | 0.0108 a | 0.0100 b | 0.5375 | 0.9903 |

[1]Expressed as octan-2-ol equivalents (mg/L). Different letters within the same row indicate significant differences.
